# Predictive analysis of the psychological state of charismatic leaders on employees' work attitudes based on artificial intelligence affective computing

**DOI:** 10.3389/fpsyg.2022.965658

**Published:** 2022-09-23

**Authors:** Yi Liu, Jaehoon Song

**Affiliations:** ^1^Department of Business Administration, Jeonju University, Jeonju, South Korea; ^2^School of Business Administration, Hebei University of Economics and Business, Shijiazhuang, Hebei, China; ^3^Department of Business Administration, Woosuk University, Wanju-Gun, Jeollabuk-do, South Korea

**Keywords:** mental state predictive analysis, staff work attitude, charismatic leadership, artificial intelligence affective computing, charismatic leaders, voice recognition technology

## Abstract

With the progress of social production, the competition for talents among enterprises is fierce, and the market often lacks capable leaders, which leads to the lack of management of enterprise employees and cannot bring more economic benefits to enterprises. Traditional leaders make subordinate employees work actively and achieve the common goal of the enterprise by exerting their own leadership characteristics and observing their subordinates, but they cannot take care of the psychological state of each employee, resulting in the employee's work efficiency is not very high. In recent years, charismatic leadership has become an important economic leader in the new era, and the theoretical spirit of charismatic leadership can well guide employees to work actively. Artificial intelligence affective computing can well identify the psychological state of the subject, and the combination of artificial intelligence affective computing and charismatic leadership can achieve effective management of employees through the predictive analysis of employees' psychological state. This paper compares the psychological state prediction analysis of employees' work attitudes between charismatic leaders based on artificial intelligence affective computing and traditional leaders through experiments. The results show that: charismatic leaders based on artificial intelligence affective computing predictive analytics can improve sensitivity to employee needs, resulting in an 8.2% increase in employee trust in leadership, a 4.4% increase in employee commitment to achieving organizational goals, and a 19.3% increase in employee job satisfaction. The psychological state prediction analysis of charismatic leaders based on artificial intelligence affective computing on employees' work attitudes can improve the work efficiency of employees and the economic benefits of enterprises.

## Introduction

Since the middle of the 20th century, the global economy has developed rapidly, the competition among enterprises is fierce, lack of competent management personnel, and there is a shortage of capable management talents. Organizations or enterprises are in urgent need of capable management talents to cope with the changing market. Therefore, charismatic leadership emerges as the times require, and the charismatic management concept is to encourage and guide employees to make positive work responses through the manager's own charisma. Charismatic leadership mainly analyzes the psychological state of subordinate employees' work attitude and makes corresponding measures according to different employee characteristics. With the development of technologies such as artificial intelligence and sentiment analysis, the results of predictive analysis of people's psychological states are becoming more and more accurate. Combining charismatic leaders with artificial intelligence emotional computing can accurately predict the work attitude of subordinate employees, and make corresponding strategies. Through the psychological state prediction analysis of the charismatic leadership of artificial intelligence affective computing on employees' work attitudes, the employees' work attitudes are more positive and the group performance of the enterprise is improved. Therefore, this paper has research significance.

The psychological state prediction analysis of charismatic leadership on employees' work attitude can make corresponding strategies for employees' psychology to improve employees' work attitude, and relevant workers have conducted in-depth research on it. Among them, Lee showed through research that the analysis of employees' psychological state by charismatic leadership can improve employees' work enthusiasm and organizational performance (Lee and Bae, 2017). Ai research pointed out that charismatic leaders and employees psychological communication can make employees take work more seriously and improve the economic efficiency of enterprises (Ai and Michelle, 2017). The results of Pramono T research show that charismatic leaders' mastery of employees' psychological state is linearly related to employees' work enthusiasm (Pramono et al., 2021). Mai-Bornu Z L compares the psychological state analysis of the two groups of leaders on the employees' work attitude, and the experiment shows that the employees have higher obedience to the charismatic leadership (Mai-Bornu, 2020). Debode J D judges the economic benefits of the company by analyzing the ability of charismatic leaders to predict the psychological state of employees' work attitudes (Debode et al., 2017). Although the psychological state prediction analysis of charismatic leaders on employees' work attitude can greatly improve the economic benefits of enterprises, there is a lack of scientific emotional computing to assist in the analysis.

With the development of artificial intelligence affective computing technology, more and more researchers use artificial intelligence affective computing technology to assist charismatic leaders to predict and analyze the psychological state of employees' work attitudes. Among them, Jamal J's research found that charismatic leaders using artificial intelligence affective computing to predict the psychological state of employees' work attitudes can improve employees' work enthusiasm to a level (Jamal and Bakar, 2017). Dartey-Baah K research shows that charismatic leadership based on artificial intelligence affective computing can better manage employees (Dartey-Baah and Addo, 2018). Putra E D pointed out that charismatic leaders use emotion recognition technology to analyze the emotional state of employees, which is more leadership (Putra and Cho, 2019). Young LM compares whether leaders use emotion recognition to analyze the emotional state of employees, and concludes that the use of emotion recognition technology can better help leaders analyze employees (Young and Gavade, 2018). Klemenc-Keti Z stated that charismatic leaders can use artificial intelligence affective computing technology to analyze employees' emotional status, which can enhance the sensitivity to employees' needs (Klemenc-Keti and Susi, 2019). Artificial intelligence affective computing technology to assist charismatic leaders can improve the accuracy of psychological state prediction and analysis of employees' work attitudes, but it is not optimal in terms of algorithms (Wu et al., 2021).

Through the application of artificial intelligence affective computing, this paper predicts and analyzes the psychological state of the charismatic leader's work attitude to employees, and compares the psychological state of the traditional leader's subordinate's work attitude. The innovations of this paper are as follows: (1) Combining artificial intelligence emotional computing technology with charismatic leadership; (2) Comparing with traditional leadership models, highlighting the advantages of charismatic leadership based on artificial intelligence emotional computing in managing subordinates.

## Artificial intelligence affective computing methods

**Figure 1:**
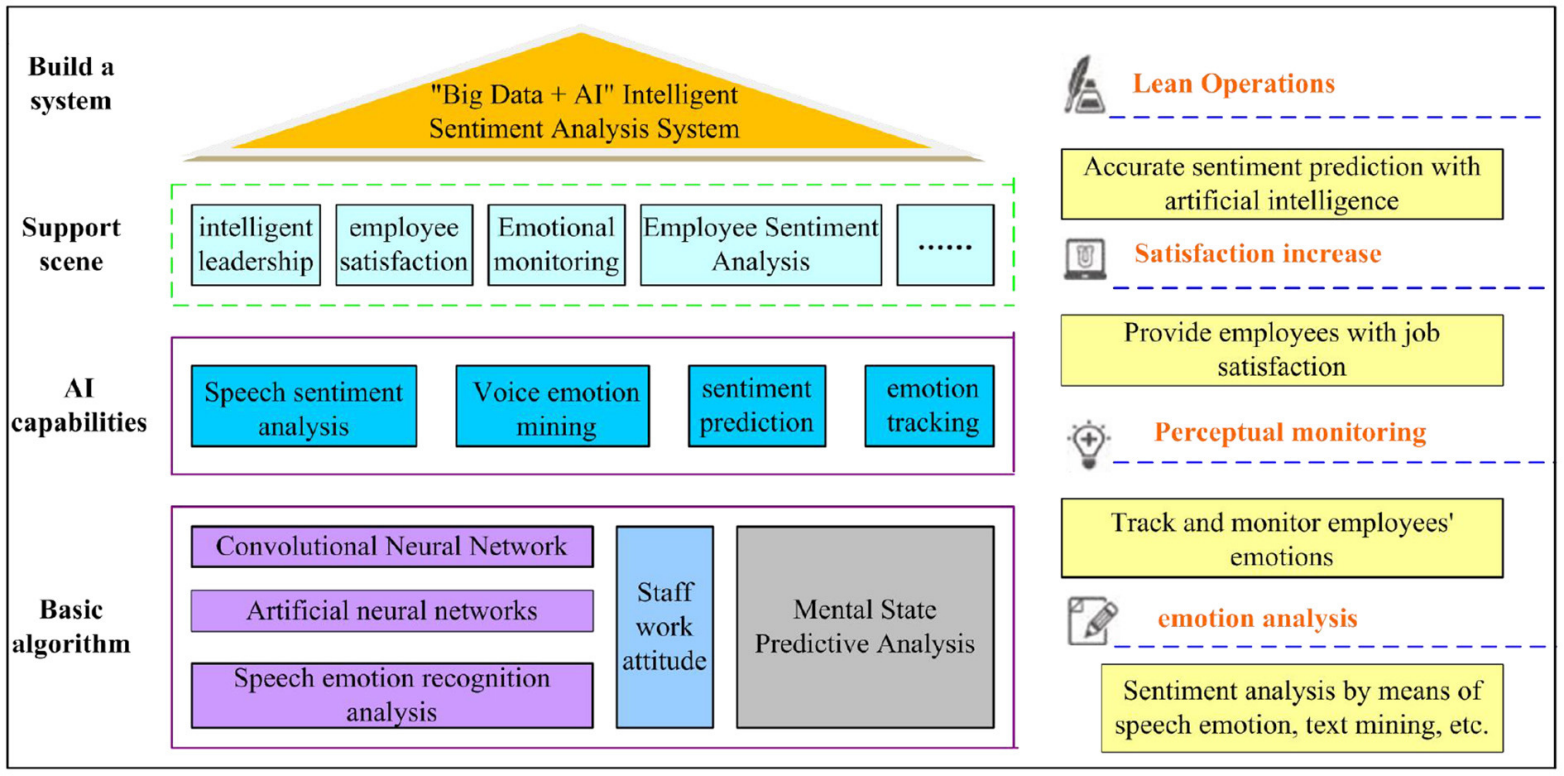
The charismatic leader based on artificial intelligence emotional computing analyzes employee sentiment map.

The combination of charismatic leadership and artificial intelligence emotional computing can improve employees' job satisfaction and improve the economic benefits of enterprises through tracking and monitoring of employees' work attitudes, emotional prediction, and emotional analysis. The underlying basic algorithms of intelligent emotional computing include speech emotion recognition algorithm, artificial neural network, convolutional neural network, etc. (Banks et al., 2017; Christianto and Smarandache, 2021). Figure 1 shows the psychological state prediction and analysis framework model of charismatic leaders based on artificial intelligence affective computing on employees' work attitudes.

### Speech emotion recognition algorithm

**Figure 2:**
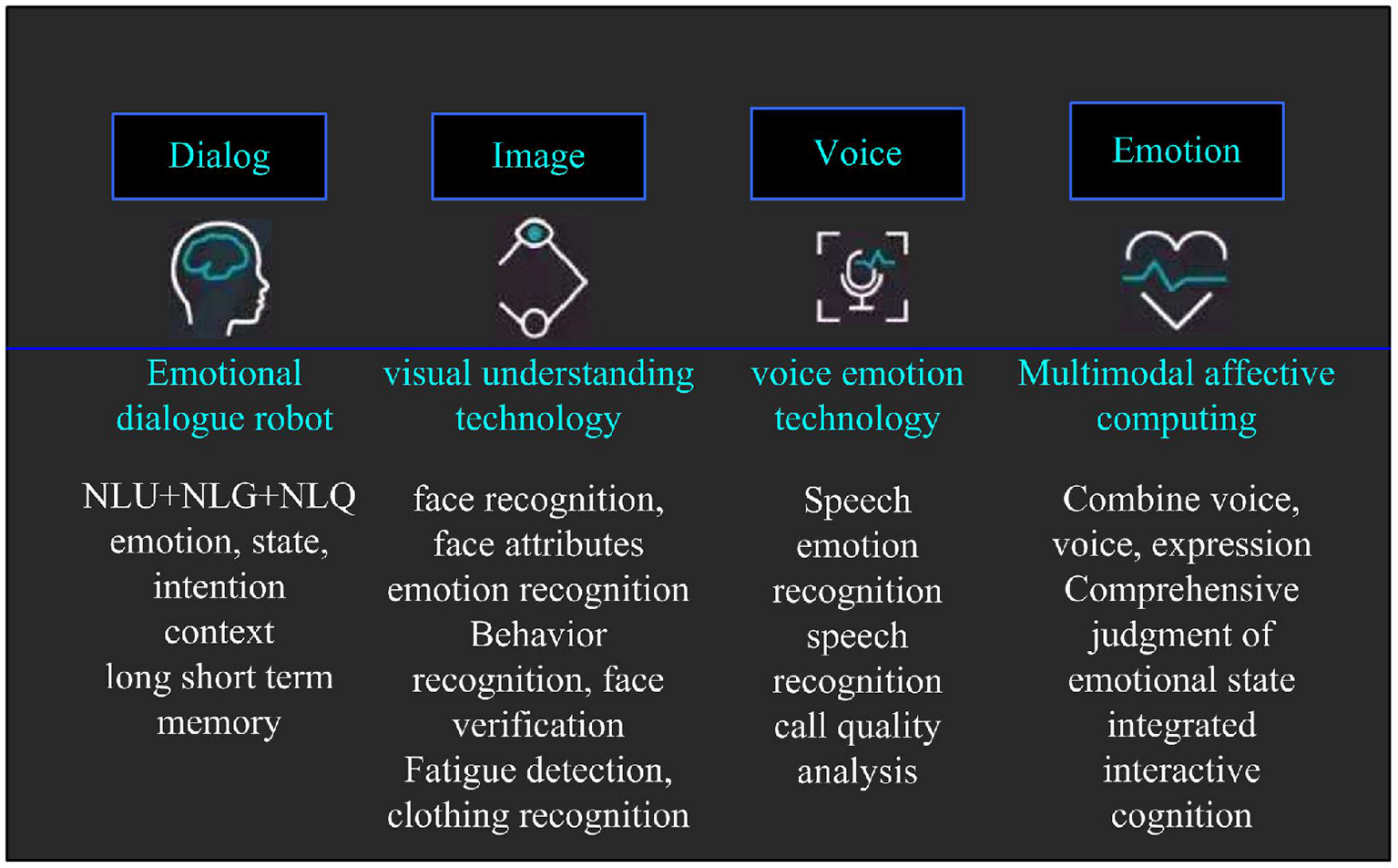
Emotion recognition model diagram.

Emotion recognition is a new direction of intelligent research, mainly through the acquisition of emotional dialogue, emotional visual understanding, emotional language understanding, and intelligent emotional computing analysis through machines (Wang et al., 2017). The emotion recognition model is shown in Figure 2. In view of the psychological state prediction analysis of the charismatic leader's work attitude to the employees in this paper, the speech emotion recognition technology is mainly use speech emotion recognition technology to identify and analyze the psychological state of employees' work attitudes.

#### Establishment of speech emotion database

Speech emotion database is the basis of speech emotion recognition and the key to the psychological state prediction analysis of employees' work attitude, so the establishment of language emotion database must be rational and extensive. The standard established by the speech emotion database is to make each speech in the emotion database can accurately express a specific emotional state (Huang et al., 2017; Ng and Law, 2021).

The voice emotion database is usually jointly created by language experts and psychology experts. Among them, a voice-over teacher with standard pronunciation is required to make emotional recordings. The emotional states of the voice emotion database are generally divided into 6 categories, including: surprise, sadness, neutrality, fear, happy, and anger. Common speech emotion databases include EMODB emotion database and Belfast emotion database (Schuller, 2018).

#### Preprocessing speech emotion signal

The voice signal sent from the population is an analog signal, and the computer's arithmetic processing is carried out in the form of binary. Therefore, to process the voice emotional signal, it is necessary to convert the voice emotional signal into a digital signal. In order to prevent the voice emotional signal In the process of conversion, signal loss occurs, and it is necessary to filter the acquisition process of the speech emotion signal, so as to obtain an accurate speech emotion signal within a certain bandwidth limit (Xun et al., 2022).

The voice quality of the digital signal voice after analog signal conversion will be degraded, and the digital signal voice needs to be preprocessed. After the preprocessing operation, the quality of the voice emotion signal will be better, which is more suitable for the analysis of the voice emotion signal. The preprocessing process of speech emotion signal includes: pre-emphasis process, speech framing and windowing, speech emotion feature extraction.

#### Pre-emphasis process

When a person is speaking, in order to accurately transmit the speech to the ear of the other party, it is necessary to vibrate the human lips and vocal cords so that the sound can be transmitted farther, but the speech signal will decrease at a certain speed in the high-frequency spectrum. As a result, there is a large gap in the speech spectrum, which is not conducive to the analysis of the speech signal. The pre-emphasis process is a frequency compensation process for the voice signal, in order to make the spectrum of the voice emotional signal smooth, which can more accurately predict the psychological state of the employee's work attitude (Papakostas et al., 2017).

The pre-emphasis process is compensated by using a first-order filter, and the pre-emphasis process is expressed as:


(1) $$\begin{array}{l} D\left(z\right)=1-\lambda z^{-1} \end{array}$$

In formula (1), it represents the pre-emphasis parameter, and the value range is [0.9, 1].

#### Voice framing and windowing

Speech emotion signal is a continuous time function, and it fluctuates greatly, so the processing method of stationary signal is obviously not suitable for the analysis of speech emotion signal. In order to better analyze the speech signal, the speech signal is divided into a small segment of speech. When the segmentation is short enough, the speech signal in a short period of time can be regarded as a stable value. Dividing each small part is called a frame, and the length of a frame is 15 ms in this paper.

However, since the language spoken by people in reality is not discontinuous, that is to say, there will be a connection between two adjacent frames, that is, the problem of overlapping of the two frames will occur, so the speech frame needs to be divided into frames. However, the framing process will cause discontinuity at the front and rear ends of the speech frame, and the more framing times, the greater the speech error. Therefore, the speech frame after framing should be processed by windowing (Swain et al., 2018).

The window function can separate the speech frames well, and the rectangular window function is expressed as:


(2) $$G(n)=\left\{ \begin{array}{l} 1,0\leq n\leq (N-1)\\ 0,other \end{array}\right.$$

The representation of the Hamming window is:


(3) $$G(n,k)=\left\{ \begin{array}{l} 1-k-k\cos(\frac{2\pi n}{N-1}),0\leq n\leq (N-1)\\ 0,other \end{array}\right.$$

In formula (3), k represents a window coefficient.

#### Emotional feature extraction of speech

In addition to the specific provisions of linguistics, the reason why people can understand the meaning of the other person's voice, there is also the unique information of the voice, which is called the feature quantity of the voice. To accurately analyze speech emotion, it is necessary to clearly understand the relationship between the characteristic parameters of speech emotion signal and emotion state. Table 1 shows the relationship between emotional state and speech feature parameters.

**Table 1:** Relationship between emotional state and speech feature parameters.

**Emotional state**	**Happy**	**Surprise**	**Neutral**	**Angry**	**Fear**	**Sad**
Speed of speech	Quick	Soon	Gentle	Quick	Soon	Slow
Spectrum curve change	Change up	Change up	Basically unchanged	Change up	Change up	Change down
Loudness	Big	Very big	Normal	Big	Very big	Smaller
Tone	High	Very high	Normal	High	Very high	Slightly lower

Analysis of the data in Table 1 shows that emotional state is closely related to language parameters such as speech rate, pitch, and loudness. The more intense the expression of speech emotion, the faster the speech rate, the higher the pitch and the greater the loudness.

In addition to the speech feature parameters in Table 1, the emotional state of speech is also related to the vibration frequency of the speech. Speech emotion feature extraction technology is usually used to extract speech rate, loudness, pitch and vibration frequency of speech emotion signal. In this paper, Mel cepstrum is used to extract speech emotion feature (Jacob, 2017).

#### Mel cepstrum

The Mel cepstral coefficient can perceive the characteristics of speech emotional signals at different frequencies. The Mel coefficient is an important indicator in the Mel cepstrum. The Mel coefficient has a nonlinear relationship with the frequency of the speech emotional signal. The Mel function is expressed as:


(4) $$\begin{array}{l} M\left(v\right)=1125\ln\;\left(1+\frac{v}{700}\right) \end{array}$$

In formula (4), v represents the frequency of the speech emotion signal, 1 n means take the logarithm.

In the process of extracting emotional features by Mel cepstrum, the process of extracting Mel coefficients by using the Mel function is as follows: the speech signal is preprocessed and then Fourier transform is performed to make the time-frequency speech signal into a frequency-domain speech signal; using a first-order filter Filtering, the filtering range is the range of human audible speech; the speech signal is processed logarithmically, and finally the speech signal is represented by trigonometric function transformation, and the change process is expressed as:


(5) $$\begin{array}{l} F_{M}\left(i\right)=\sqrt{2/N}\overset{d}{\underset{i=1}{\sum }}m_{d}\cos\left(\left(d-0.6\right)i\pi /d\right) \end{array}$$

#### Short-term energy

Short-term energy is the energy attached to language emotional signals in a very short period of time. The short-term energy is related to the vibration of the sound. Usually, the more intense the emotion, the greater the short-term energy. For example, the short-term energy of fear or surprise is generally than the short-term energy of grief. The short-term energy pass is used to distinguish between unvoiced and voiced speech (Alghifari et al., 2018).

The formula for short-term energy is:


(6) $$\begin{array}{l} E_{x}=\overset{N-1}{\underset{n=0}{\sum }}s_{x}^{2}\left(n\right) \end{array}$$

In formula (6), *s*_*x*_(*n*) represents the speech emotion signal.

The short-term jitter energy of the voice signal is:


(7) $$\begin{array}{l} E_{k}=\frac{\frac{1}{N-1}\overset{N-1}{\underset{x-1}{\sum }}|E_{x}-E_{x+1}|}{\frac{1}{N}\overset{N}{\underset{x=1}{\sum }}E_{x}} \end{array}$$

In formula (7), *E*_*k*_ represents the short-term jitter energy of the speech signal.

The linear regression equation of short-term energy is expressed as:


(8) $$\begin{array}{l} E_{g}=\frac{\overset{N}{\underset{x=1}{\sum }}x\cdot E_{x}-\frac{1}{N}\overset{N}{\underset{x=1}{\sum }}x\cdot \overset{N}{\underset{x=1}{\sum }}E_{x}}{\overset{N}{\underset{x=1}{\sum }}x^{2}-\frac{1}{N}\left(\overset{N}{\underset{x=0}{\sum }}x\right)} \end{array}$$

In formula (8), *E*_*g*_ represents a linear regression coefficient of short-term energy.

The short-term zero-crossing rate is a way to detect the presence or absence of sound at both ends of the voice signal, and the short-term zero-crossing rate is the number of times the voice signal frame crosses the time axis. The short-term zero-crossing rate is expressed as:


(9) $$\begin{array}{l} Q_{x}=\frac{1}{2}\overset{N-2}{\underset{n=0}{\sum }}|sgn\left[s_{x}\left(n\right)\right]-sgn\left[s_{x}\left(n-1\right)\right]| \end{array}$$

In formula (9), *s*_*x*_ is the speech emotion signal frame, and *Q*_*x*_ is the short-term zero-crossing rate, sgn stands for symbolic function.

sgn [x] is a mathematical symbolic function, which is specifically expressed as:


(10) $$sgn[x]=\left\{ \begin{array}{l} 1,(x\geq 0)\\ -1,(x<0) \end{array}\right.$$

### Artificial neural network algorithm

The artificial neural network is a process that imitates the human brain to process information. The artificial neural network is composed of many neuron nodes. It has high memory and learning ability and can accurately predict the psychological state of the employee's work attitude (Alanis, 2018).

#### Artificial neurons

**Figure 3:**
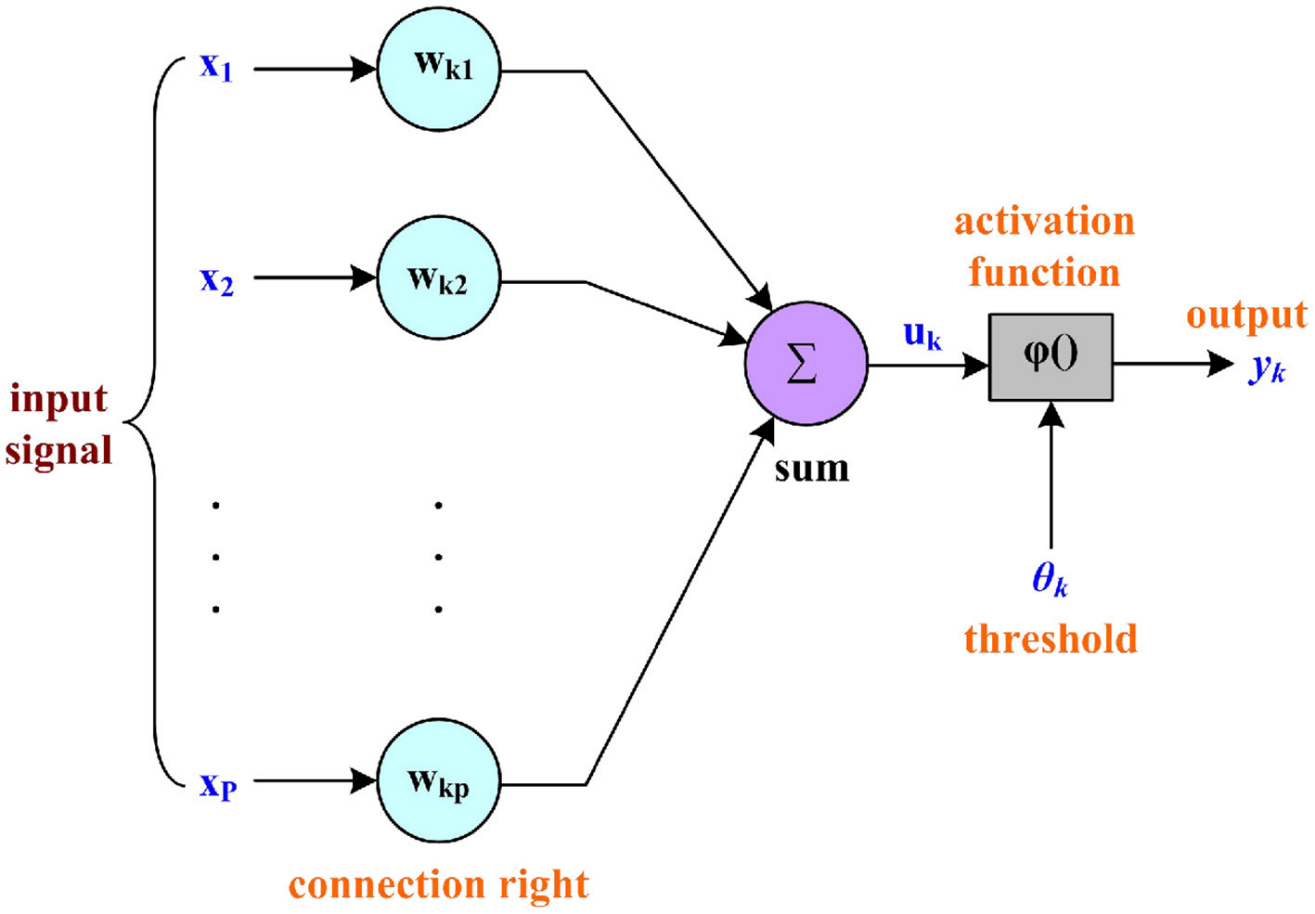
Structure diagram of artificial neuron.

Artificial neuron is the most basic processing unit of artificial neural network. The structure of artificial neuron is shown in Figure 3.

The function of the artificial neuron is expressed as:


(11) $$\begin{array}{l} net=\overset{p}{\underset{i=1}{\sum }}w_{ki}x_{i} \end{array}$$

In Equation (11), net represents artificial neuron processing.


(12) $$\begin{array}{l} y_{k}=\phi \left(net\right) \end{array}$$

When ϕ() is a threshold function, the output is expressed as:


(13) $$\begin{array}{l} y_{k}=sgn\left(\overset{p}{\underset{i=1}{\sum }}w_{ki}x_{i}-\theta_{k}\right) \end{array}$$

In Equation (13), θ_*k*_ represents the threshold of the k^th^ neuron.

#### Topological structure of artificial neural network

An artificial neural network is composed of a large number of neuron nodes connected by a specific connection method. The connections between neurons are different, and the resulting artificial neural network structures are also different. The structure of artificial neural network is mainly divided into: forward network and feedback network (Li et al., 2017).

**Figure 4:**
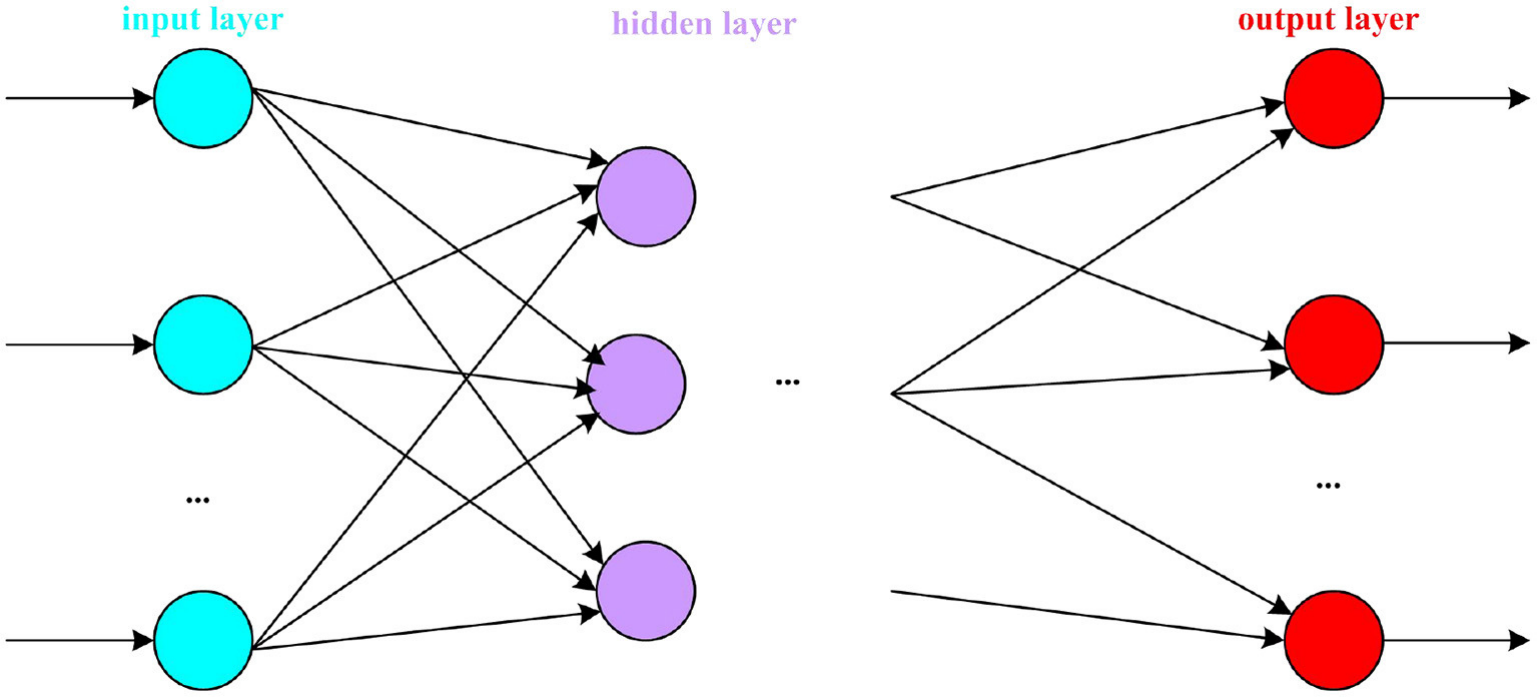
Forward network topology diagram.

The forward network structure from left to right is: input layer, hidden layer, and output layer. The transmission of information must be a step from the input layer to the hidden layer and then to the output layer. The topology of the forward network is shown in Figure 4.

**Figure 5:**
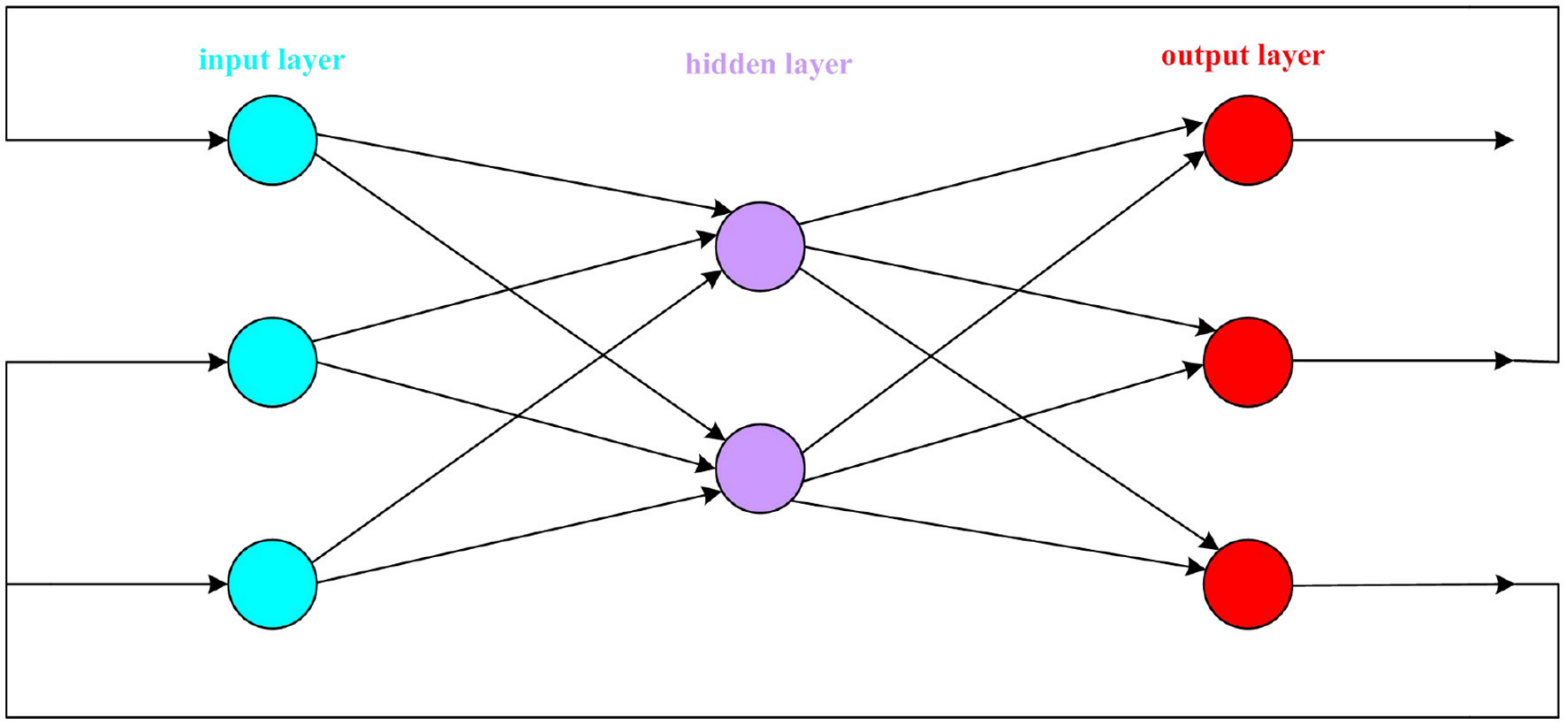
Feedback network topology diagram.

The feedback network is also a three-layer structure. A feedback mechanism is added on the basis of the forward network. The information still flows from the input layer to the hidden layer to the output layer. The error information will be transmitted from the output layer to the hidden layer in the form of feedback. Input layer, where error is the difference between the expected output and the actual output (Safa et al., 2018). The network topology of the feedback network is shown in Figure 5.

#### The learning process of artificial neural network

Assuming that neuron a receives the output of neuron b, if the excitation of neuron a and neuron b both increase, the network structure will increase the connection weight between the two neurons a and b. The learning process of artificial neural network is expressed as:


(14) $$\begin{array}{l} \Delta w_{i}=\lambda yx_{i} \end{array}$$

In formula (14), λ represents the learning rate, and Δ*w*_*i*_ represents the change amount of the i-th connection weight.

### Convolutional neural network algorithm

**Figure 6:**
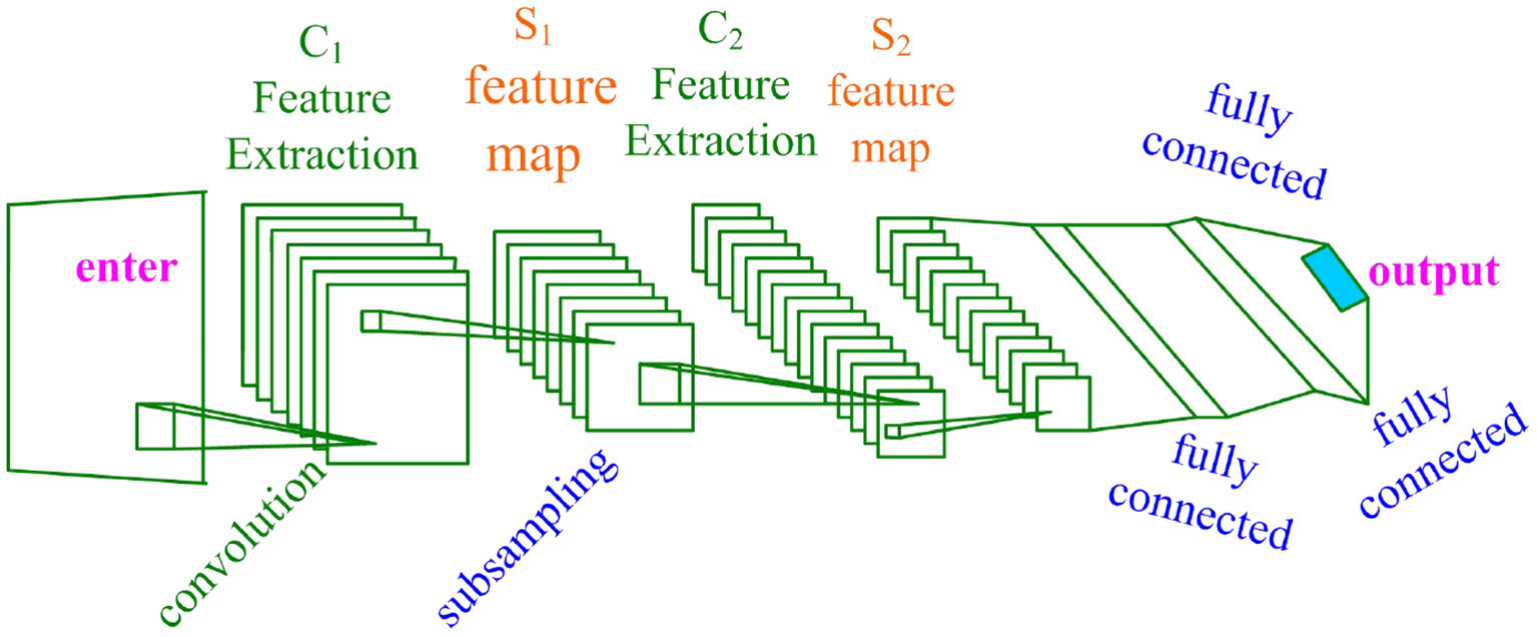
Convolutional neural network topology diagram.

Convolutional neural network is a network mechanism based on artificial neural network. Convolutional neural network has the characteristics of deep learning and is widely used in analysis of emotion recognition, image recognition, speech recognition and so on. The network topology of the convolutional neural network is shown in Figure 6.

As can be seen from Figure 6, the convolutional neural network consists of a convolutional layer, a sub-sampling layer, and a fully connected layer. The convolutional neural network is a multi-layer induction model with strong learning capabilities (Kruthiventi et al., 2017).

The convolutional data is subjected to the bias operation and then processed by the activation function to obtain the output data. The convolutional layer formula is expressed as:


(15) $$s_{r}^{t}=f(\sum_{i\in M_{r}}s_{i}^{t-1}*k_{ir}^{t}+g_{r}^{t})$$

In formula (15), $s_{r}^{t}$ represents the r-th feature data of the t-th convolutional layer, and $k_{ir}^{t}$ represents the weight of the r-th feature data representing the i-th input value of the t-th convolutional layer.

The subsampling layer is to sample and separate the input data and reduce the data dimension. The sampling dimension reduction process of the subsampling layer is expressed as:


(16) $$\begin{array}{l} s_{r}^{i}=f\left(D_{r}^{t}d\left(s_{r}^{t-1}\right)+g_{r}^{t}\right) \end{array}$$

In formula (16), *d*() represents the sampling dimension reduction function, and $D_{r}^{t}$ represents the weighting parameter.

There is also a process of backpropagation in the learning process of convolutional neural networks. Take the reverse derivative of the fully connected layer:


(17) $$\begin{array}{l} \frac{\partial Q}{\partial w\left(t\right)}=\delta^{\left(t\right)}{\left(s^{\left(t-1\right)}\right)}^{T} \end{array}$$

(18) $$\begin{array}{l} \frac{\partial Q}{\partial b\left(t\right)}=\delta^{\left(t\right)} \end{array}$$

In formula (17) and formula (18), t represents the current convolutional layer, and t-1 represents the previous convolutional layer.

During backpropagation, the transfer process from the convolutional layer t-1 to the t layer is:


(19) $$\begin{array}{l} s_{i}^{\left(t\right)}=f\left(\overset{N_{t-1}}{\underset{m=1}{\sum }}C2\left(s_{m}^{\left(t-1\right)},K_{im}^{\left(t\right)}\right)+g_{r}^{t}\right) \end{array}$$

19)

In formula (19), C represents the convolution calculation process, and *N*_*t*−1_ represents the number of features obtained in the t-1 layer.

Experiment and analysis on the psychological state prediction analysis of charismatic leaders based on artificial intelligence affective computing on employees' work attitudes.

### Experimental data

#### Sample data

In order to better analyze the psychological state prediction analysis of charismatic leaders and traditional leaders on employees' work attitudes, this paper analyzes charismatic leaders with artificial intelligence emotional computing, while traditional leaders only rely on daily data and experience such as personal attendance. The selection of experimental samples is very important. Poor selection of experimental samples can easily lead to invalid experimental comparisons. The sample data needs to cover the entire sample as much as possible.

This experiment will select 20 companies and divide the employees and leaders of the companies into two groups, namely the charismatic leadership group and the traditional leadership group. An extrinsic indicator of psychological state predictive analysis that affects leaders' attitudes toward employees' work. Table 2 shows the psychological state predictive analysis indicators that affect leaders' attitudes toward employees' work.

**Table 2:** Predictive analysis indicators of psychological states that affect leaders' attitudes toward employees' work.

**Evaluation indicators**	**Charismatic leadership based on artificial intelligence affective computing**	**Traditional leadership**
Employee's trust in leadership	83%	78%
Employee's sense of mission to achieve organizational goals	84%	78%
Employee satisfaction with work	82%	82%
Sensitivity of leaders to employee needs	76%	84%
Leader's vision to motivate employees	48%	62%
Leaders care about their subordinates	56%	68%
Average	71.5%	75.3%

From the analysis of the data in Table 2, it can be seen that through the statistics of external indicators that can affect the psychological state prediction analysis of leaders' attitude toward employees, the average influence of the six indicators in Table 2 on charismatic leaders and traditional leaders is: 71.5 and 75.3%. All have a high degree of influence, and the correlation analysis can be carried out on the six indicators in Table 2 and the psychological state prediction analysis of leaders' attitude toward employees' work.

#### Correlation analysis of samples

When selecting the psychological state prediction and analysis indicators for evaluating leaders' attitude toward employees' work, it is necessary to carry out a correlation analysis between the above-mentioned influencing indicators and the psychological state prediction analysis of leaders' attitude toward employees' work (Yu et al., 2021). The correlation analysis of the samples is to prevent the failure of the experiment due to improper selection of samples. The correlation analysis of the samples is to clarify the main characteristics of the objects to be studied, and it is easier to observe which indicators and leaders' attitudes toward employees' work. The correlation degree of psychological state prediction analysis, the results of correlation analysis of the above six influence indicators are shown in Table 3.

**Table 3:** Correlation analysis of the psychological state prediction analysis of leaders' attitude toward employees' work.

**Number of sample groups**	**Evaluation indicators**	**Relevance**
1	Employee's trust in leadership	0.248
2	Employee's sense of mission to achieve organizational goals	0.214
3	Employee satisfaction with work	0.241
4	Sensitivity of leaders to employee needs	0.237
5	Leader's vision to motivate employees	0.016
6	Leaders care about their subordinates	0.044

In Table 3, the correlation index of employees' trust in charismatic leaders is 0.248, and the index with the smallest correlation is the leader's vision incentive to employees, and the correlation is 0.016. Compared with the first four indicators, the correlation between indicators 5 and 6 is much smaller, so only the first four indicators need to be followed up for comparative analysis.

#### Validity analysis of samples

In order to test the first four indicators in Table 3 to evaluate the psychological state prediction analysis of charismatic leaders based on artificial intelligence affective computing on employees' work attitudes and traditional leaders' psychological state prediction analysis of employees' work attitudes. The experiment needs to analyze the validity of the samples with high correlation degree. In this experiment, the 4-fold cross-validation method is used for the verification analysis (Dizdarevic et al., 2020). The validity analysis results of 4-fold cross-validation are shown in Table 4.

**Table 4:** Effectiveness analysis result table.

**Impact indicator**	**Charismatic leadership based on artificial intelligence affective computing**	**Traditional leadership**
Employee's trust in leadership	82.4%	74.2%
Employee's sense of mission to achieve organizational goals	80.8%	76.4%
Employee satisfaction with work	82.4%	82.2%
Sensitivity of leaders to employee needs	86.8%	76.2%
Average	83.1%	77.3%

The data in Table 4 shows that in the predictive analysis of the psychological state of charismatic leaders based on artificial intelligence affective computing on employees' work attitudes, the highest effectiveness indicator is the leader's sensitivity to employee needs, and the average effectiveness of the four indicators is 83.1%.; The highest validity index in the psychological state prediction analysis of traditional leaders' attitude toward employees is employee satisfaction with work, and the average validity of the four indexes is 77.3%. Therefore, the above four indicators can be compared and analyzed between charismatic leaders and traditional leaders based on artificial intelligence affective computing to judge the psychological state prediction analysis experiment of employees' work attitude.

### Comparative experiment of charismatic leadership and traditional leadership based on artificial intelligence affective computing

#### Employee's trust in leadership

**Figure 7:**
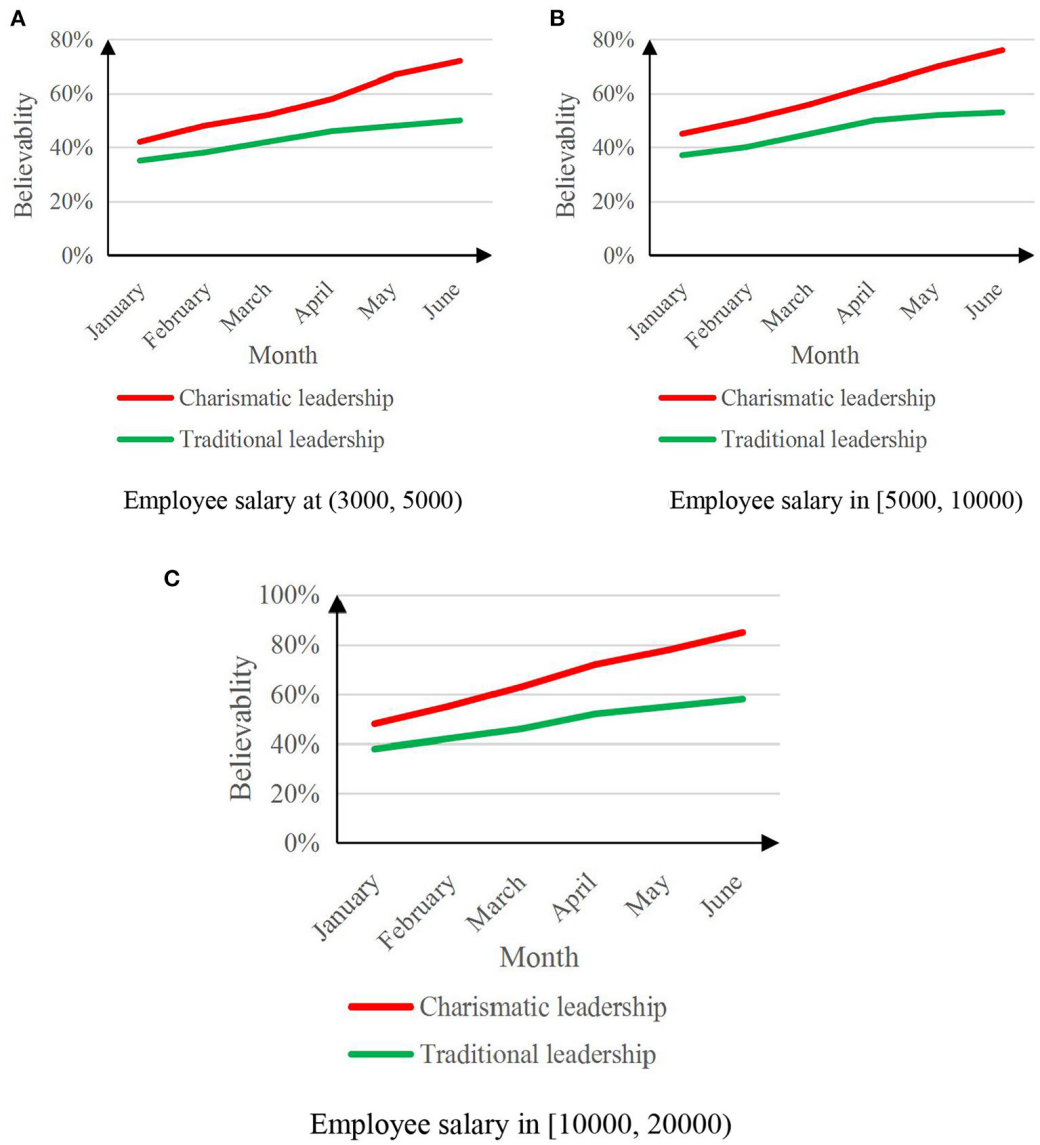
Graph of employee trust in two types of leaders. **(A)** Employee salary at (3,000, 5,000). **(B)** Employee salary in [5,000, 10,000). **(C)** Employee salary in [10,000, 20,000].

The psychological state prediction analysis of the leader's attitude toward the employee's work can help understand the employee's work emotion, make timely adjustment strategies, and increase the employee's trust in the leader. In order to compare employees' trust in the two types of leaders, the experiment will select employees with different salary levels to conduct the experiment, among which 50 people with salaries between (3,000, 5,000), 50 people with salaries between [5,000, 10,000], [10,000, 20,000] between 50 people. The experimental period was set to 6 months, and employees' trust in leaders was surveyed every month. Figure 7 shows the results of employees' trust in leaders based on the psychological state prediction analysis of two leaders' attitudes toward employees' work.

Data reflects: Predictive analysis of the psychological state of charismatic leaders based on artificial intelligence emotional computing on employees' work attitudes will improve employees' trust in leaders, and the higher the employee's salary, the more obvious the increase in trust, and the greater the employee's confidence in charismatic leaders. The highest level of trust in leadership is 85%.

#### The sense of mission of employees to achieve organizational goals

**Figure 8:**
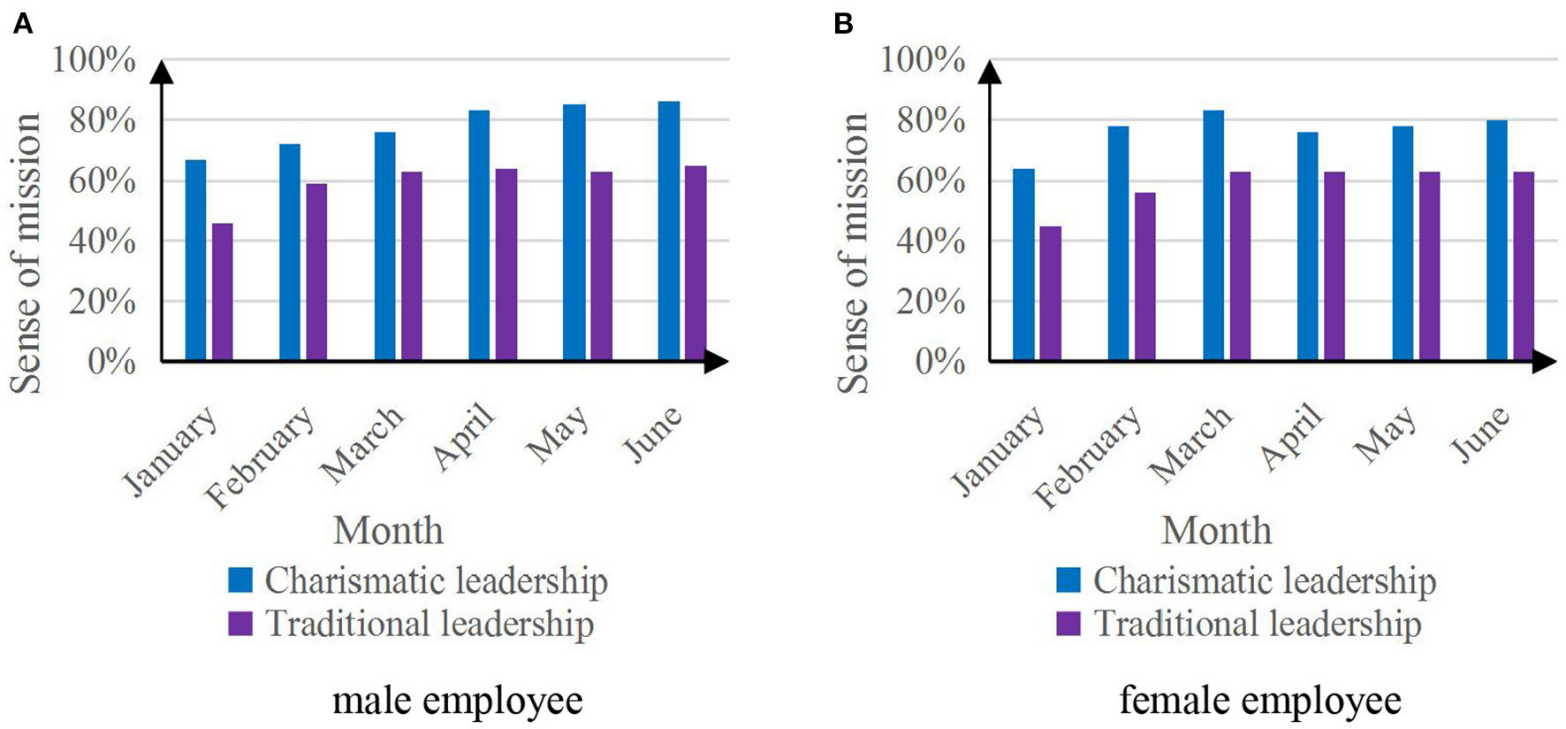
Sense of mission map of employees to achieve organizational goals. **(A)** Male employee. **(B)** Female employee.

The sense of mission of employees to achieve organizational goals is an important indicator to measure the psychological state predictive analysis of leaders' attitudes toward employees. Since male and female employees may interfere with the achievement, the experimental employees are separated by gender. Figure 8 shows the results of employees' sense of mission to achieve organizational goals based on the psychological state prediction analysis of the two leaders' attitudes toward employees' work.

The data in Figure 8 reflect that the psychological state prediction analysis of charismatic leaders based on artificial intelligence affective computing on employees' work attitudes can improve employees' sense of mission in achieving organizational goals, and male employees are more affected by charismatic leadership than female employees. The charismatic leader's sense of mission for employees to achieve organizational goals can reach up to 86%, and the sense of mission of traditional leaders for employees to achieve organizational goals can reach up to 65%.

#### Employee satisfaction with work

**Figure 9:**
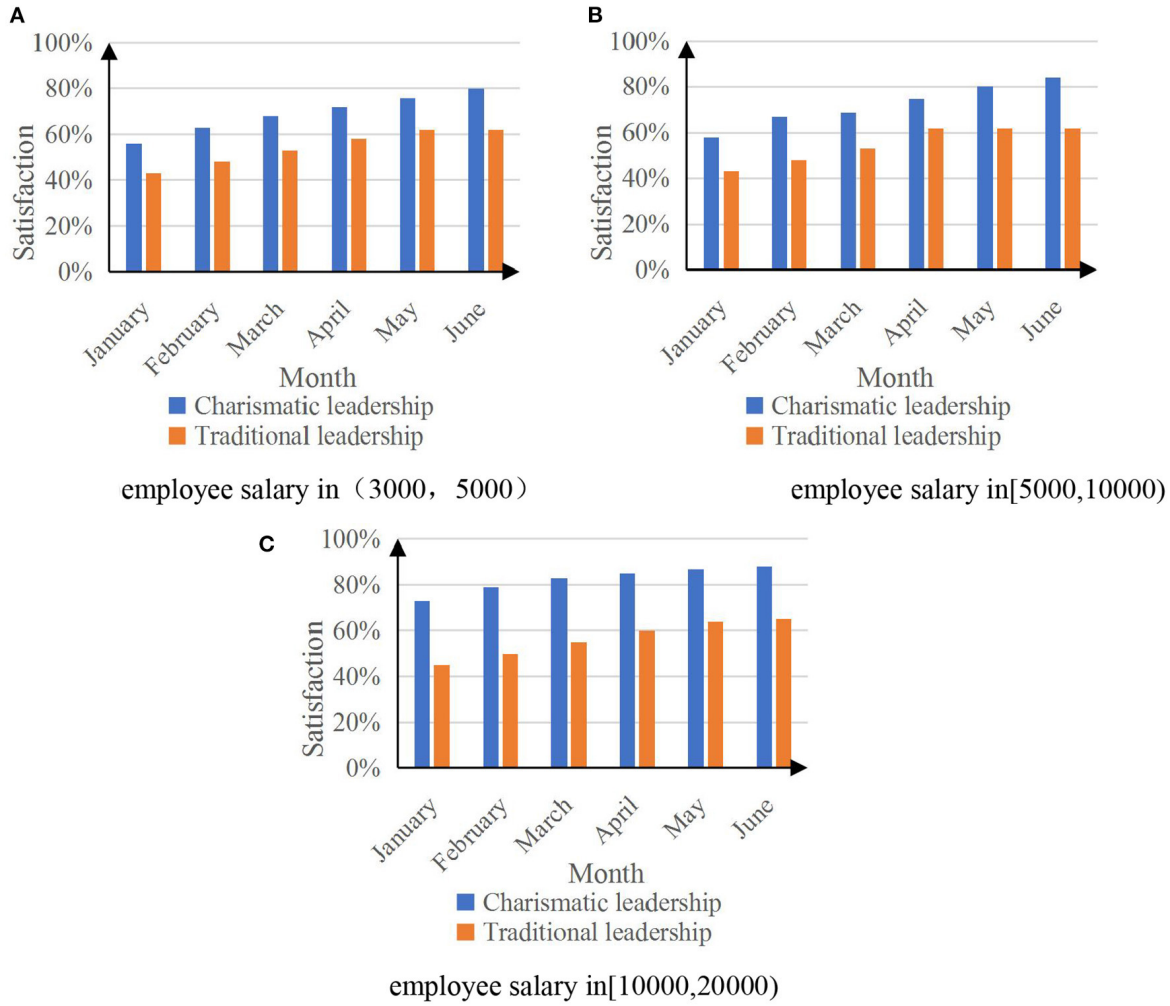
Employee's job satisfaction graph. **(A)** Employee salary in (3,000, 5,000). **(B)** Employee salary in (5,000, 10,000). **(C)** Employee salary in (10,000, 20,000).

The employee's job satisfaction is the dominant result of the leader's psychological state prediction analysis of the employee's work attitude. A 6-month job satisfaction survey was conducted on three tiers of salary employees. Figure 9 shows the results of employees' job satisfaction based on the psychological state prediction analysis of the two leaders' attitudes toward employees' work.

The data in Figure 9 reflect that the psychological state prediction analysis of charismatic leaders on the work attitudes of three salary employees can improve employees' job satisfaction, and the satisfaction rate can reach up to 88%. The psychological state prediction analysis of traditional leaders on the three types of salary employees' work attitudes has little effect on improving employees' job satisfaction.

#### Sensitivity of leaders to the needs of employees

**Figure 10:**
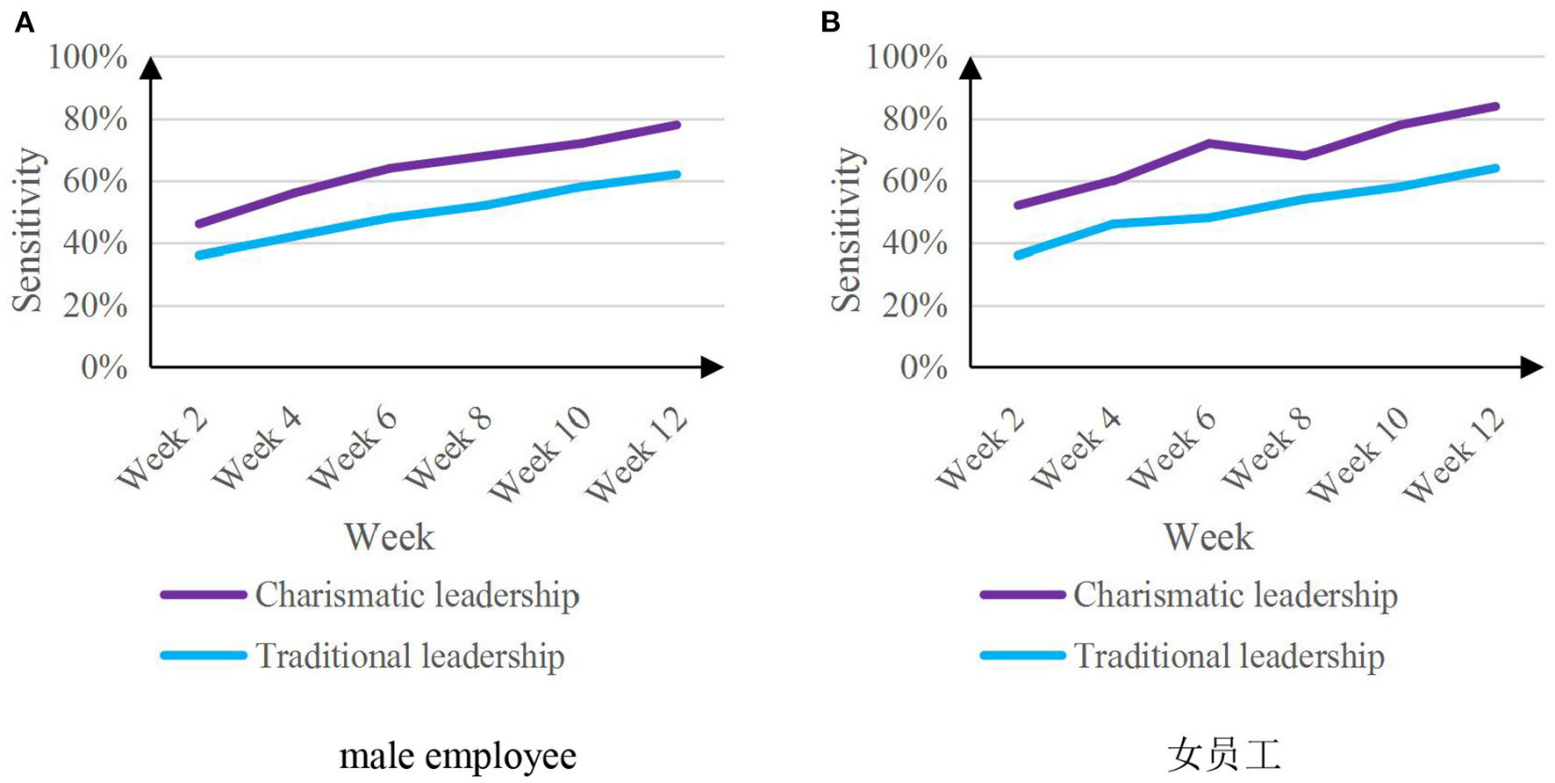
Sensitivity map of leaders to employee needs. **(A)** Male employee. **(B)** 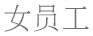.

Predictive analysis of leaders' psychological state of employees' work attitudes can help leaders better understand employees' needs. The experiment will investigate leaders' sensitivity to needs of employees of different genders. The experiment will be conducted every 2 weeks for a period of 12 weeks. Figure 10 shows the sensitivity results of two kinds of leaders' psychological state prediction analysis on employees' work attitude to employees' needs.

From the data analysis in Figure 10, it can be seen that the charismatic leaders are more sensitive to the needs of male and female employees than the traditional leaders during the test period. The average sensitivity of 50.3%.

### Experimental analysis

Through a multi-faceted comparison of the psychological state prediction analysis of the two leaders' attitudes toward employees' work, the experimental results show that the charismatic leadership based on artificial intelligence affective computing has an important effect on employees' trust in leaders, employees' sense of mission to achieve organizational goals, and employees' commitment to work. Satisfaction, leadership sensitivity to employee needs are better than traditional leadership. Table 5 shows the multi-faceted average results of the psychological state prediction analysis of the specific two kinds of leaders' attitude toward employees' work.

**Table 5:** The average data comparison table of the psychological state prediction analysis of the two leaders' attitude toward employees' work.

**Compare items**	**Charismatic leadership based on artificial intelligence affective computing**	**Traditional leadership**
Employee's trust in leadership	61.1%	45.9%
Employee's sense of mission to achieve organizational goals	73.3%	59.4%
Employee satisfaction with work	74.6%	55.3%
Sensitivity of leaders to employee needs	66.5%	50.3%

## Discussion

Traditional leaders cannot accurately predict and analyze the psychological prediction of their employees, which leads to a series of problems of low work efficiency of employees such as low enthusiasm for work and insufficient trust in leaders, which leads to low economic benefits for enterprises. Charismatic leaders can use artificial intelligence affective computing technology to predict and analyze the psychological state of employees' work attitudes and improve the economic benefits of enterprises.

## Conclusions

Through the comparative analysis of 4 aspects of the psychological state of employees' work attitudes between charismatic leaders and traditional leaders based on artificial intelligence affective computing, the experiment shows that: (1) charismatic leaders and traditional leaders based on artificial intelligence affective computing The average job satisfaction is: 74.6 and 55.3%, respectively; the sense of mission of employees under charismatic and traditional leadership to achieve organizational goals is: 73.3 and 59.4%, respectively. (2) Charismatic leaders can use artificial intelligence emotional computing to analyze the psychological state of employees' work attitudes, which can make leaders more sensitive to employees' needs, and make employees trust leaders 15.2% more than traditional leaders. The psychological state prediction analysis of charismatic leaders and traditional leaders based on artificial intelligence affective computing on employees' work attitudes can make subordinate employees trust leaders more, improve work efficiency, and increase group performance. However, at present, the accuracy of artificial intelligence emotional computing's prediction of the psychological state of employees' work attitudes cannot reach a very accurate level. Therefore, improving the accuracy of psychological state prediction of employees' work attitude will be the direction of future research.

## Data availability statement

The original contributions presented in the study are included in the article/supplementary material, further inquiries can be directed to the corresponding author.

## Author contributions

YL: writing-original draft preparation. JS: editing data curation and supervision. Both authors contributed to the article and approved the submitted version.

## Conflict of interest

The authors declare that the research was conducted in the absence of any commercial or financial relationships that could be construed as a potential conflict of interest.

## Publisher's note

All claims expressed in this article are solely those of the authors and do not necessarily represent those of their affiliated organizations, or those of the publisher, the editors and the reviewers. Any product that may be evaluated in this article, or claim that may be made by its manufacturer, is not guaranteed or endorsed by the publisher.
